# The Nursing Implementation Complexity Tool for Digital Technology Implementation in Nursing: Design Science Research Study

**DOI:** 10.2196/105536

**Published:** 2026-09-30

**Authors:** Stefan Walzer, Erik Farin-Glattacker, Christophe Kunze

**Affiliations:** 1 Care & Technology Lab, Furtwangen University, Furtwangen, Baden-Wurttemberg, Germany; 2 Section of Health Care Research and Rehabilitation Research, Institute of Medical Biometry and Statistics, University of Freiburg, Medical Faculty and Medical Center, Hugstetter Str. 49, Freiburg, 79106, Germany, 49 0761 270-74430

**Keywords:** digital health, digital nursing technologies, implementation science, Nonadoption, Abandonment, Scale-Up, Spread, and Sustainability, NASSS framework, implementation complexity, design science research, nursing, implementation reflection

## Abstract

**Background:**

Implementing digital health technologies is challenging because implementation is shaped by interacting technical, organizational, professional, and contextual factors. Although implementation frameworks such as the Nonadoption, Abandonment, Scale-Up, Spread, and Sustainability (NASSS) framework support understanding this complexity, translating them into practical tools for routine implementation remains difficult.

**Objective:**

This study aimed to develop and evaluate the Nursing Implementation Complexity Tool (NICT), a theory-informed implementation reflection tool designed to operationalize the NASSS framework and support implementation-related reflection and decision-making for digital technology implementation in nursing.

**Methods:**

This Design Science Research study combined 4 iterative development phases with a convergent mixed methods evaluation. The development of the NICT was informed by an umbrella review, 3 mixed methods case studies, and the German adaptation of the NASSS Complexity Assessment Tool (NASSS-CAT-D). Following iterative development and expert review, the NICT was evaluated through workshops with nursing professionals, prospective nursing leaders, and nursing students. A summative evaluation included an online survey (28 participants completed the questionnaire) and think-aloud interviews (8 participants). Quantitative data were analyzed descriptively, qualitative data were analyzed using qualitative content analysis, and findings were integrated during interpretation.

**Results:**

The NICT comprises 3 complementary components: a guideline, a visual canvas, and a pocket card organized around 4 reflection domains. Across the formative and summative evaluations, participants consistently perceived the NICT as a useful and well-structured implementation reflection tool. In the summative evaluation, 24 of 26 (92.3%) participants agreed that the NICT had a logical structure, and 24 of 26 (92.3%) agreed that it supported structured reflection on implementation-related factors. Qualitative findings showed that the NICT facilitated discussion of implementation determinants, stakeholder perspectives, and contextual influences while also highlighting the importance of practical guidance, examples, and facilitation to support its application.

**Conclusions:**

The NICT supports nursing professionals and other implementation stakeholders in recognizing, discussing, and addressing implementation complexity through structured reflection. Rather than reducing complexity itself, it facilitates implementation-related reflection and informed decision-making. Beyond its application in nursing, this study illustrates how conceptual frameworks for understanding implementation complexity can be translated into structured, practice-oriented reflection tools for digital health implementation.

## Introduction

### Background

Digital technologies encompass a broad range of applications in nursing care, including telehealth services, clinical decision support systems, sensor-based monitoring technologies, and care robots. These technologies are increasingly regarded as an important strategy to improve care delivery, enhance patient safety, and support health care professionals in their daily work [1,2]. A growing body of evidence indicates that many innovations, even when demonstrating positive effects, fail to transition from project-based implementation to sustained routine use [3-7]. The reasons for this are multifactorial and reflect the complexity of implementing digital health technologies in real-world health care settings [8,9].

Successful implementation of digital health technologies requires collaboration among multiple implementation stakeholders. Within this landscape, nurses play a pivotal role. As the largest professional group in health care and primary users of many digital technologies in clinical care, they are central to determining the success or failure of implementation efforts [10]. However, digital technologies are frequently introduced without sufficient consideration of clinical workflows, professional values, care processes, or user needs, which can hinder their adoption and integration into routine practice [8,11]. Moreover, nurses operate at the intersection of patient care, interprofessional collaboration, and organizational processes, making them key stakeholders in adapting technologies to local contexts and everyday clinical routines.

To better understand and address the complexity of technology implementation in health care, several theoretical frameworks have been developed. Among them, the Nonadoption, Abandonment, Scale-up, Spread, and Sustainability (NASSS) framework [4] provides a comprehensive approach to analyzing implementation processes across 7 interrelated domains. In an overview of evaluation frameworks applicable to digital nursing technologies, NASSS was identified as one of the most comprehensive and broadly applicable approaches [12]. Other frameworks, such as the Consolidated Framework for Implementation Research (CFIR) [3], similarly emphasize the importance of contextual factors and multilevel interactions. Together, these frameworks have substantially advanced implementation science by providing structured approaches to understanding implementation complexity and identifying determinants that influence implementation success.

While these frameworks provide valuable conceptual guidance, practical tools such as the NASSS Complexity Assessment Tools (NASSS-CATs) have been developed to facilitate their application [5]. However, translating implementation theory into routine health care practice remains challenging. Our previous translation and contextual adaptation of the German version of the NASSS-CAT (NASSS-CAT-D) highlighted the need for clearer guidance, greater contextualization, and more practice-oriented support for routine nursing implementation [13]. Existing implementation frameworks often require substantial methodological expertise, making them difficult to operationalize in everyday implementation practice [9,14]. In nursing care, this includes consideration of profession-specific factors such as clinical workflows, professional responsibilities, and care priorities.

As a result, implementation stakeholders often lack accessible, practice-oriented tools that operationalize implementation theory to support structured reflection and implementation planning while accounting for the inherent complexity of implementation processes [5]. This need is particularly evident in nursing care, where digital technologies are frequently implemented in highly complex clinical environments and nurses play a central role in their adoption and integration into routine practice.

### Aim and Research Question

To address this gap, the aim of this study was to develop and iteratively evaluate the Nursing Implementation Complexity Tool (NICT), a theory-informed, practice-oriented implementation reflection tool designed to support implementation planning for digital nursing technologies.

The study addresses the following research questions:

- How can implementation theory and implementation experiences be translated into a practice-oriented implementation reflection tool to support implementation planning for digital nursing technologies?
- How do nursing professionals perceive the usefulness, comprehensibility, and applicability of the NICT?
- To what extent does the NICT support structured reflection on barriers and facilitators relevant to the implementation of digital nursing technologies?
- What strengths, limitations, and areas for improvement do nursing professionals identify regarding the use of the NICT in practice?

## Methods

### Overview

This manuscript was guided by the GUIDED (Guidance for Reporting Intervention Development Studies in Health Research) [15], SRQR (Standards for Reporting Qualitative Research) [16], and GRAMMS (Good Reporting of a Mixed Methods Study) recommendations [17].

### Design

The development and evaluation of the NICT followed a Design Science Research (DSR) approach [18]. The study was guided by the interplay between the relevance cycle, the rigor cycle, and the design cycle, thereby integrating practice needs, existing scientific knowledge, and iterative artifact development (Figure 1). The relevance cycle drew on empirical insights from nursing practice to identify implementation challenges, user requirements, and contextual factors. The rigor cycle ensured theoretical grounding through the NASSS framework [4].

**Figure 1.**
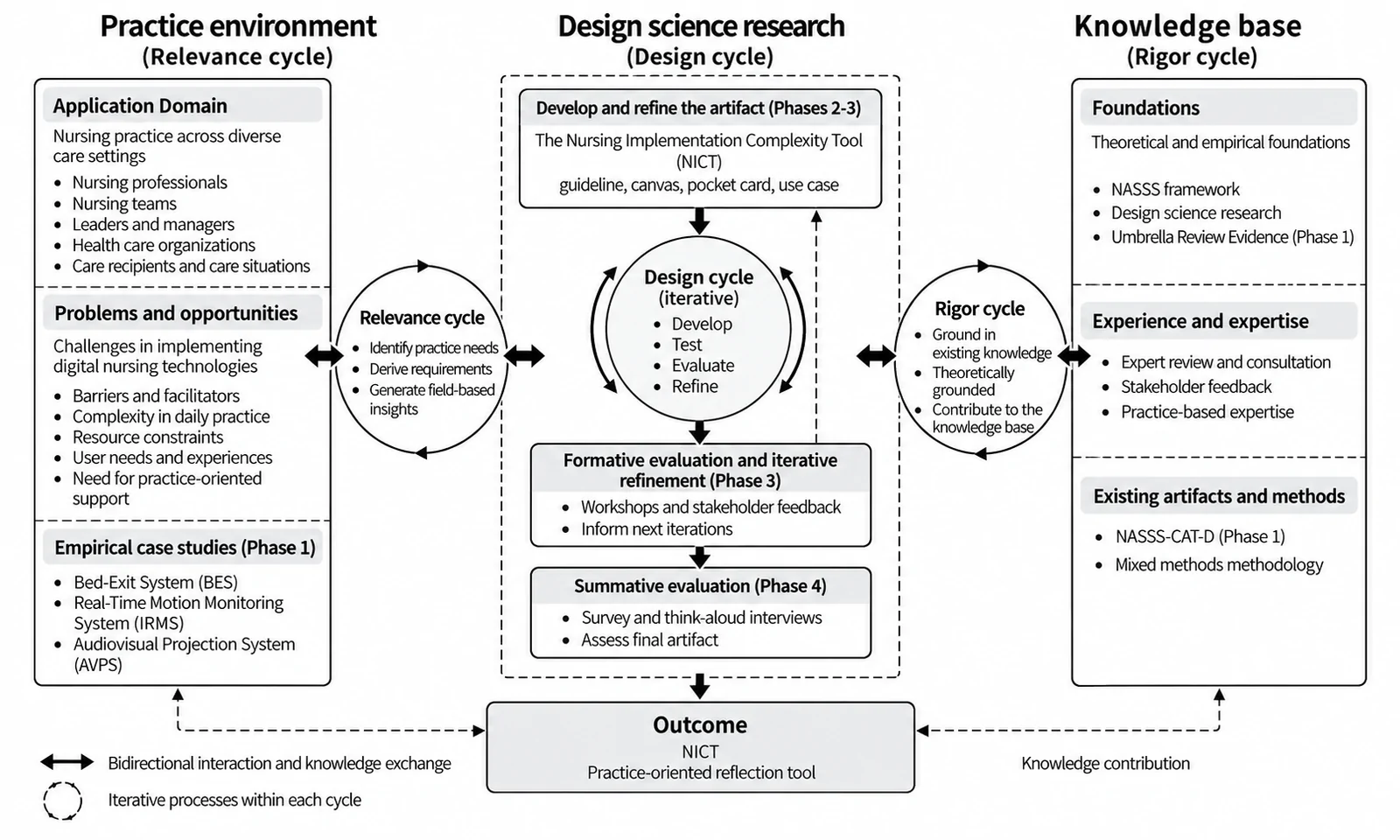
Design Science Research process underlying the development and evaluation of the NICT. NASSS: Nonadoption, Abandonment, Scale-Up, Spread, and Sustainability.

Within the overall DSR framework, the development and evaluation of the NICT employed a convergent mixed methods approach [19]. Qualitative and quantitative data were collected across the different development phases to generate complementary evidence for the iterative development, refinement, and evaluation of the NICT. Qualitative evidence was given greater interpretive priority, while quantitative data provided complementary descriptive evidence.

Within this overarching DSR framework, the design cycle was operationalized through 4 interrelated phases:

- Phase 1: conceptual grounding
- Phase 2: tool development
- Phase 3: formative evaluation
- Phase 4: summative evaluation

Phases 1 and 2 primarily focused on the conceptualization, development, and refinement of the NICT, whereas phases 3 and 4 aimed to generate insights into its usability, perceived relevance, and contextual requirements across different user groups and application settings.

### Phase 1: Conceptual Foundation

#### Overview

This phase aimed to establish a theoretically and empirically grounded foundation for the iterative development of the NICT. Following a design-oriented and iterative research approach, multiple complementary data sources were systematically integrated to identify relevant implementation domains, underlying mechanisms, and contextual requirements for the implementation of digital nursing technologies.

The phase integrated evidence from three complementary sources: (1) a systematic umbrella review examining barriers, facilitators, and influencing factors related to the implementation and adoption of digital technologies in nursing; (2) empirical case studies conducted at the University Medical Center Freiburg; and (3) the translated and contextually adapted NASSS-CAT-D as an implementation support tool.

An overview of the iterative development of the NICT across all 4 DSR phases and the contribution of the different evidence sources is provided in Multimedia Appendix 1.

#### Data Sources and Integration

The central objective of this phase was the iterative synthesis of findings across these 3 sources. Using a triangulation approach informed by principles of framework synthesis [20], findings from the umbrella review, empirical case studies, and the NASSS-CAT-D were iteratively compared and integrated to identify converging and complementary patterns and to map these onto overarching implementation domains relevant to nursing practice. The synthesis was conducted by the primary researcher and iteratively discussed within the research team to enhance interpretive rigor and reduce subjective bias.

The umbrella review [8] synthesized existing evidence on factors influencing the implementation and adoption of digital technologies in nursing across different care settings, identifying 52 implementation determinants across the 6 NASSS domains, including barriers related to training, workload, technological confidence, professional roles, autonomy, and privacy, as well as facilitators such as leadership support, organizational culture, and targeted training. The empirical foundation comprised 3 mixed methods case studies investigating nurses’ experiences with a bed-exit system [21], a real-time motion monitoring system [22], and an audiovisual projection system (S Walzer, MSc, unpublished data, 2026). Across these studies, empirical data comprised interviews, workshops and focus groups, participant observations, and nursing staff survey responses. These studies provided in-depth insights into adoption dynamics, user acceptance, and contextual constraints. Although conducted in acute care settings, the findings are considered analytically transferable to comparable health care contexts [23].

In parallel, the NASSS-CAT-D [13], adapted from the original NASSS-CAT [5], served as an additional source for operationalizing implementation complexity in the nursing context. The synthesis of these 3 sources informed the identification of key implementation domains and provided the conceptual foundation for the development of the NICT in phase 2.

### Phase 2: Tool Development

#### Overview

Phase 2 focused on the iterative development and refinement of the NICT between August 2023 and July 2024. The process was led by the primary researcher in close collaboration with an interdisciplinary research team and practice-oriented stakeholders.

Building on the findings from phase 1^13^, the NICT was developed through structured working sessions, regular research meetings, and iterative feedback loops.

Insights from interviews, observations, focus groups, and workshops were continuously integrated into the iterative design process to refine the content, structure, wording, and usability of the NICT. An initial draft of the tool was reviewed by practice-based experts regarding its completeness, clarity, relevance, and practical applicability. Their feedback, together with additional input on ethical and legal aspects, informed subsequent revisions. This iterative process resulted in a refined version of the NICT, which was subsequently subjected to formative evaluation in phase 3.

#### Translation Into Tool Components

The development process focused on translating the identified implementation determinants into practically usable formats. This included the iterative condensation, abstraction, and operationalization of the findings into structured reflection domains, guiding questions, and application examples.

The initial version of the NICT was primarily developed for acute inpatient nursing care, reflecting the empirical evidence base and case studies that informed its development. However, the underlying implementation domains and reflection questions were intentionally formulated to be applicable across different nursing care settings. While specific examples, terminology, and contextual considerations may require adaptation to local workflows and organizational structures, the core domains related to care recipients, technology, users, and context are considered transferable beyond acute care.

The resulting implementation reflection tool comprised three complementary components:

- A narrative guideline document providing the rationale for the tool, outlining its theoretical and empirical foundations, and offering instructions for using the canvas and pocket card.
- A visual canvas to support collaborative reflection, discussion, and documentation.
- A pocket card, summarizing core principles and guiding questions.

#### Design Principles and Accessibility

The design followed principles of usability, accessibility, and knowledge translation, aiming to operationalize implementation theory into a practical implementation reflection tool that is understandable and actionable for nursing professionals. Visual structuring was used to support cognitive processing, facilitate interdisciplinary exchange, and reduce complexity in decision-making processes. To promote transparency and further development, the first version of the tool was made publicly available in German via an institutional open-access repository under a Creative Commons (CC BY 4.0) license [24].

### Phase 3: Testing

#### Overview

Phase 3 focused on the formative evaluation and iterative refinements of the NICT through participatory workshop formats. The aim was to explore its usability, comprehensibility, and perceived practical relevance across different user groups and application contexts while generating empirical evidence to inform subsequent refinements of the artifact.

#### Design and Procedure

The workshops were designed to simulate real-world implementation planning and collaborative reflection related to the implementation of digital nursing technologies. Between 2023 and 2025, the tool was applied in three distinct settings: (1) a workshop at a nursing conference (n=48), (2) a workshop with prospective nurse managers (n=21), and (3) a workshop with nursing students (n=63). Each workshop included an introductory session on digital innovation in nursing, followed by small-group exercises (4‐6 participants per group) applying the NICT to specific implementation scenarios. The scenarios were based on digital technologies presented through a combination of case examples, slide-based information, and hands-on demonstrations of the actual devices. Example technologies included telepresence robotics and real-time motion monitoring systems.

#### Data Collection

Data were collected from three complementary sources: (1) written materials generated during the small-group exercises and plenary presentations (eg, completed canvas templates and group summaries), (2) postworkshop feedback collected through an anonymous digital evaluation survey administered via Particify, and (3) field notes recorded by the first author during and immediately after each session. The combination of qualitative and quantitative data sources was intended to provide complementary evidence regarding the usability, practical applicability, and further refinement of the NICT. The Particify survey included both closed-ended items assessing participants’ perceptions of the workshop, the usability of the NICT, and the perceived relevance of the content, as well as open-ended questions inviting suggestions for improvement. Field notes focused on participants’ interactions with the tool, group dynamics, implementation challenges, and recurring themes identified during the discussions.

#### Analysis and Iteration

Qualitative and quantitative data were initially analyzed separately according to their respective methodological approaches and subsequently integrated during interpretation to identify recurring themes, improvement needs, and opportunities for further refinement of the NICT. The integrated findings were discussed in regular research team meetings to enhance the credibility of the interpretations, and decisions regarding revisions were reached through consensus among the authors.

### Phase 4: Summative Evaluation

#### Overview

Phase 4 aimed to conduct a summative evaluation of the refined NICT to assess its usability, comprehensibility, and perceived usefulness, while generating additional evidence to inform the final refinement of the artifact.

#### Design and Data Collection

A mixed-methods approach was applied, combining a standardized online questionnaire with qualitative think-aloud interviews [25]. The combination of quantitative and qualitative methods was intended to provide complementary evidence regarding both users’ overall perceptions of the NICT and their interaction with the tool during practical application. The online survey was conducted in 2026 with nursing researchers, nursing professionals, and leaders (n=28 completed questionnaires). Participants were recruited via professional networks and direct contact with relevant nursing organizations and institutions. Item generation was informed by the findings and design refinements from the preceding DSR phases (Multimedia Appendix 2). To ensure content validity and clarity, the instrument was iteratively refined through internal review and pilot testing within the research context. The pretest involved three colleagues with expertise in nursing research, who completed the questionnaire and provided feedback. Based on their comments, several items were reworded for improved comprehensibility.

The survey assessed key aspects of the tool, including usability, clarity, and perceived usefulness in supporting implementation-related reflection and implementation planning, based on a standardized questionnaire comprising 12 items using a 6-point Likert scale (ranging from “strongly disagree” to “strongly agree,” with an additional “unable to assess” option), complemented by 7 open-ended questions. To complement these findings and explore emerging patterns from the survey responses, think-aloud interviews (n=8) were conducted with survey participants who had indicated their willingness to participate in a follow-up interview. Participants were asked to use the tool while verbalizing their thoughts, enabling detailed insights into cognitive processes, interaction patterns, and potential usability challenges during application.

#### Analysis

Quantitative data from the online survey were analyzed descriptively to summarize user ratings and identify general trends regarding usability and perceived usefulness. Qualitative data from the think-aloud interviews were analyzed using qualitative content analysis [26] to explore user experiences, difficulties, and potential improvements in greater depth. Quantitative and qualitative findings were subsequently integrated during interpretation using a convergence coding approach adapted from Farmer et al [27]. Findings were compared across data sources and classified as agreement, partial agreement, silence, or dissonance to obtain a comprehensive understanding of users’ perceptions, interaction patterns, and refinement needs of the NICT (Multimedia Appendix 3).

### Ethical Considerations

The study was conducted in accordance with the Declaration of Helsinki and approved by the ethics committee of Furtwangen University (approval number: 25‐122). Participation in all study components was voluntary, and participants were informed about the study procedures and data protection measures. Workshop-based evaluations were conducted in educational and professional contexts; no identifying data were collected, and participation was considered as implied consent. For the online survey and think-aloud interviews, informed consent was obtained prior to participation. All data were analyzed in an anonymized form. Ethical procedures for the empirical studies informing phase 1 are reported in the respective publications.

## Results

The results are presented according to the 4 study-specific phases used to operationalize the design cycle within the overarching DSR approach.

The final versions of the NICT components are provided in Multimedia Appendices 4-6, and an exemplary use case is presented in Multimedia Appendix 7.

### Conceptual Foundation (Phase 1)

Phase 1 aimed to identify and synthesize implementation determinants relevant to the introduction of digital technologies in nursing care and to derive design requirements for NICT.

Findings from the preceding umbrella review, empirical case studies, and the NASSS-CAT-D revealed a range of interrelated determinants operating at the microlevel, mesolevel, and macrolevel. Key barriers consistently identified across studies included misalignments between technology manufacturers and nursing staff regarding perceived challenges and appropriate solutions, difficulties integrating technologies into existing nursing workflows, inadequate training provision, limited organizational readiness, and insufficient consideration of nursing-specific requirements within existing implementation tools.

To operationalize these findings into actionable design principles, the identified determinants were systematically mapped to the domains of the NASSS framework and iteratively discussed within the research team. This synthesis process resulted in 4 preliminary reflection areas: care recipients, health professionals, technology, and organizational context. Ethical and legal considerations were conceptualized as cross-cutting dimensions relevant across all domains.

Together, these integrated findings informed the design requirements for the NICT. Key requirements included the need for a practical, user-centered, and context-sensitive tool that supports structured reflection, facilitates interdisciplinary communication, and enables the consideration of implementation complexity across multiple levels.

The outcome of this phase was a conceptual framework comprising 4 reflection areas and cross-cutting ethical and legal considerations, which served as the conceptual foundation for the iterative development of the initial NICT prototype in phase 2.

### Tool Development (Phase 2)

#### Initial Tool Development

The resulting instrument comprises three complementary components: a structured guide with reflection prompts, a visually designed canvas for team-based application, and a pocket card to facilitate low-threshold use in everyday practice.

Together, these components make implementation complexity more explicit and support systematic, multiperspective reflection across domains. Each domain is represented as a quadrant of the canvas and linked through visual connections, emphasizing interaction and iterative reflection rather than linear decision-making. Short guiding questions on the pocket card support structured reflection and implementation planning. The NICT is intended to support implementation stakeholders involved in planning, implementing, facilitating, and evaluating digital technologies in nursing and is designed for both individual and collaborative use, for example in workshops, project meetings, or educational settings.

#### Expert-Based Refinement

To further refine the initial prototype, an expert review was conducted at the University Medical Center Freiburg involving nine stakeholders from nursing management, advanced nursing practice, clinical ethics, information technology, and data protection. Overall, participants perceived the NICT as a useful and well-structured approach to supporting the implementation of digital technologies in nursing care. In particular, the pocket card and the canvas were valued for facilitating perspective-taking, identifying relevant stakeholders, and ensuring that important aspects are not overlooked. At the same time, participants highlighted the need for clearer guidance on how to apply the different tool components throughout implementation processes. Additional practical examples, step-by-step instructions, and digital formats were suggested to enhance usability. Furthermore, the accompanying materials were perceived as overly academic, and participants recommended simplifying the language and improving the integration of visual elements. All feedback from the expert review was systematically reviewed and incorporated into the iterative refinement of the NICT. Revisions focused on improving clarity, usability, and practical applicability through simplified language, enhanced guidance, and the inclusion of application examples.

The outcome of this phase was version 1 of the NICT, incorporating the refinements derived from the expert review and providing the foundation for the formative evaluation conducted in phase 3.

### Testing (Phase 3)

Version 1 of the NICT was applied in 3 workshop-based settings involving nursing professionals, prospective nursing leaders, and nursing students.

Across all settings, participants perceived the NICT as a useful and practice-oriented tool for supporting reflection on complex implementation processes. The completed canvas templates, participant feedback, and facilitator field notes consistently indicated that the NICT supported users in identifying and reflecting on relevant implementation determinants and considering interactions between care recipient needs, technology characteristics, professional perspectives, and organizational conditions. Participants particularly valued the tool’s ability to facilitate communication and establish a shared understanding among different stakeholder groups. In leadership-oriented settings, the NICT supported discussions on roles, responsibilities, stakeholder involvement, and resource allocation. In educational contexts, participants reported that the tool fostered systems thinking and helped link theoretical concepts with practical implementation challenges.

Across all workshops, the NICT was described as clear, easy to use, and beneficial for structuring discussions around digital technology implementation in nursing care. At the same time, the testing phase highlighted important prerequisites for the effective use of the NICT in workshop settings. Participants emphasized the need for sufficient time for group work, reflection, and presentation of results, as well as smaller group sizes and appropriate working spaces. Furthermore, the workshops demonstrated that detailed descriptions of the implementation context are essential for meaningful reflection. Participants were better able to engage with the NICT when implementation scenarios included comprehensive information about the care setting, target population, existing workflows, organizational conditions, and the intended use of the technology. More detailed scenarios facilitated perspective-taking and enabled participants to better anticipate potential barriers, facilitators, and implementation requirements.

Overall, the testing phase demonstrated the perceived usefulness, usability, and adaptability of version 1 of the NICT across diverse nursing-related settings. While no substantial modifications to the NICT itself were considered necessary, the integrated findings informed refinements to the workshop design and facilitation process, including adjustments to time allocation, group size, scenario development, and instructional guidance.

### Summative Evaluation (Phase 4)

#### Quantitative Findings

A total of 28 participants completed the survey and were included in the summative evaluation. Data collection took place between December 2025 and January 2026. As respondents were able to skip individual items, the number of valid responses varied across survey questions. Participant characteristics are presented in Table 1.

**Table 1.** Sample characteristics.

Characteristic	Value, n (%)^a^
Professional role	
Research staff or scientific personnel	14 (66.7)
Nursing professionals (including combined roles with project or leadership)	3 (14.3)
Project management or administrative or executive roles	4 (19.0)
Years of professional experience	
<5	5 (23.8)
5 to <10	6 (28.6)
10 to <20	3 (14.3)
≥20	7 (33.3)
Experience with technology implementation	
None	3 (13.6)
Low	4 (18.2)
Moderate	9 (40.9)
High	6 (27.3)
Primary care setting	
Long-term care (institutional)	3 (16.7)
Outpatient care (including counseling)	4 (22.2)
Acute care or hospital setting	5 (27.8)
Mixed settings (outpatient and inpatient)	3 (16.7)
Other (eg, ethical consultation and unspecified)	3 (16.7)

a Percentages are based on valid responses (missing and incomplete responses excluded).

Overall, participants rated the NICT positively across items assessing usability, clarity, and perceived usefulness. Participants reported that the tool supports structured reflection on the implementation of digital nursing technologies and facilitates a comprehensive consideration of relevant implementation factors. High levels of agreement were observed for the logical structure of the tool (24/26, 92.3%; mean 4.50, SD 0.65) and its ability to support reflection on barriers and success factors (24/26, 92.3%; mean 4.42, SD 0.90). Similarly, a large proportion of participants indicated that the tool provides a comprehensive overview (20/26, 76.9%; mean 4.00, SD 0.98) and helps to structure complexity (19/25, 76.0%; mean 4.04, SD 0.93). The pocket card was rated as a particularly helpful element supporting initial engagement (22/26, 84.6%; mean 4.54, SD 0.76), and most participants expressed a preference for a digital version of the tool (20/24, 83.3%; mean 4.46, SD 0.78). Detailed item-level ratings are presented in Table 2.

**Table 2.** Participant ratings of the usability, usefulness, and applicability of the Nursing Implementation Complexity Tool.

Item	Agree, n/N (%)^a^	Mean (SD)	Min–Max
Helpful for implementation	20/27 (74.1)	4.00 (1.14)	1‐5
Would have used earlier	12/18 (66.7)	3.67 (1.24)	1‐5
Easy to understand	19/28 (67.9)	3.96 (1.07)	1‐5
Easy to apply	16/25 (64.0)	3.64 (0.95)	2‐5
Logical structure	24/26 (92.3)	4.50 (0.65)	3‐5
Comprehensive overview	20/26 (76.9)	4.00 (0.98)	2‐5
Supports reflection	24/26 (92.3)	4.42 (0.90)	1‐5
Canvas structures complexity	19/25 (76.0)	4.04 (0.93)	2‐5
Pocket card useful	22/26 (84.6)	4.54 (0.76)	3‐5
Suitable for other settings	19/22 (86.4)	4.32 (1.09)	1‐5
Suitable for teaching	15/21 (71.4)	3.90 (1.18)	1‐5
Digital version useful	20/24 (83.3)	4.46 (0.78)	3‐5

a Likert-scale responses were coded from 1 (“strongly disagree”) to 5 (“strongly agree”). “Cannot judge” responses and missing values were excluded. Percentages represent the proportion of agreement (scores 4‐5) based on valid responses per item.

In contrast, ratings related to ease of use and application were more heterogeneous. While a majority agreed that the tool is easy to understand (19/28, 67.9%; mean 3.96, SD 1.07) and apply (16/25, 64.0%; mean 3.64, SD 0.95), these items showed lower agreement rates and greater variability. Perceptions of suitability for teaching were also positive overall but more heterogeneous (15/21, 71.4%; mean 3.90, SD 1.18). Suitability for other care settings received higher agreement (19/22, 86.4%; mean 4.32, SD 1.09).

#### Qualitative Findings From Survey Free-Text Responses

##### Overview

The open-ended survey responses provided additional insights into participants’ perceptions of the NICT, particularly regarding its components, usability, applicability across contexts, and support needs. Across all free-text questions, responses indicated a generally positive perception of the tool’s conceptual value, combined with recurring suggestions for simplification, clearer guidance, and additional practical support materials. Participation rates across the free-text items (FT) were high and relatively consistent, with responses ranging from 17 to 24 per item (FT01: n=23; FT02: n=24; FT03: n=24; FT04: n=22; FT05: n=20; FT06: n=22; FT07: n=17), indicating strong engagement with the qualitative components of the survey.

##### Helpful Elements of the Tool (FT01)

Participants most frequently highlighted the pocket card and the canvas as particularly useful elements. These were described as providing structure, supporting orientation, and facilitating reflection on relevant aspects of technology implementation. The combination of different formats (guidance text, canvas, pocket card) was also perceived as beneficial, allowing engagement at different levels of detail. One participant stated, “The pocket card provides a quick overview and prompts reflection on key aspects that should be considered during implementation.”

The pocket card was frequently described as translating abstract concepts into accessible questions, while the canvas was valued for visually structuring complex relationships and supporting group discussions. Some participants noted that the practical use of these elements would require clearer guidance.

##### Perceived Complexity and Usability Challenges (FT02)

Many respondents reported challenges related to complexity, length, and practical applicability. The guidance document was described as extensive, academically phrased, and not always aligned with everyday nursing practice. One participant stated:

*Overall, I find the recommendation too complex and not sufficiently practice-oriented. It shows well what a canvas is, but not how to actually create one with a team.*

Participants also described limited clarity regarding how the different components of the tool relate to each other, particularly the transition from theoretical background to practical application. In addition, the broad target group and level of abstraction were reported as making it difficult to determine the intended use in specific situations. Several responses referred to the canvas primarily as a starting point rather than a comprehensive implementation guide.

##### Application Across Implementation Phases (FT03)

Participants reported that the NICT would primarily be used in early phases of implementation, particularly during project initiation and planning. It was described as supporting overview, identification of relevant factors, and decision-making processes. One participant stated, “I would primarily use it at the beginning of a project to gain an overview and decide whether a technology should be implemented.”

At the same time, some respondents described potential use across different phases of the implementation process, including evaluation or situations in which implementation processes stagnate. For later phases, additional tools for detailed project management were considered necessary.

##### Transferability to Other Care Settings (FT04)

Most participants considered the NICT to be transferable across different care settings, including acute care, long-term care, rehabilitation, and ambulatory care. This was attributed to its broad and flexible structure. One participant stated, “The recommendation is broadly formulated; the specific setting is defined by the project itself.”

At the same time, respondents emphasized that application in different contexts would require adaptation. Differences in organizational structures, patient populations, and care processes—particularly in ambulatory settings—were mentioned as relevant factors.

##### Adaptation Needs for Other Settings (FT05)

Participants identified several areas where adaptations would be necessary for use in different contexts. These included setting-specific examples, adjustments to guiding questions, and stronger consideration of contextual factors such as workflows, time constraints, and organizational characteristics. One participant stated, “The guiding questions should be adapted to the specific organization to better support transfer into practice.”

Particular challenges were described for ambulatory care settings, including limited contact time and decentralized structures.

##### Need for Additional Support Materials (FT06)

A consistent theme across responses was the need for additional support materials. Frequently mentioned were example canvases, case studies, short training formats, and digital tools. One participant stated, “An example canvas would be especially helpful to better understand how detailed the individual fields should be filled out.”

Further suggestions included instructional videos, digital versions of the canvas and pocket card, and tools for collaborative work. At the same time, some participants emphasized the importance of maintaining simplicity and avoiding excessive complexity.

##### Additional Remarks and Overall Impressions (FT07)

Participants expressed appreciation for the overall concept, structure, and design of the NICT, including the integration of ethical and legal aspects. One participant stated, “Very appealing design and wording—it encourages the reader to continue reading.”

At the same time, respondents referred to the need for clearer target group definitions, more concrete guidance for application, and improved alignment between conceptual and practical elements.

Overall, the free-text responses confirmed the quantitative findings while providing additional insights into usability challenges, contextual adaptation needs, and requirements for practical implementation support.

### Qualitative Findings of the Interviews

#### Overview

The think-aloud interviews provided in-depth insights into how participants interacted with the NICT and how they made sense of its structure and content during use. Participants went through the NICT together with the interviewer and verbalized their thoughts along the way. The sample comprised participants from nursing practice (n=4), including individuals in leadership roles, as well as research associates in nursing science (n=4). Overall, the findings confirmed the perceived value of the tool as a structured reflection instrument, while at the same time revealing tensions related to usability, clarity, and practical applicability. Four main themes were identified: (1) structuring and making complexity visible, (2) tension between conceptual depth and practical usability, (3) need for guidance and facilitation, and (4) flexible use across implementation phases.

#### Structuring and Making Complexity Visible

Across interviews, participants described the NICT as supporting the structuring of complex implementation processes and making relevant dimensions more visible during use. The tool prompted consideration of multiple perspectives, including care recipient-related, technological, user-related, and contextual factors. One participant stated, “You are forced to think about aspects that you might otherwise overlook.” (Int_2).

Participants further reported that the tool helped to externalize implicit knowledge and to integrate different viewpoints within a shared reflection process. This was particularly evident in descriptions of team-based use, where the canvas was seen as facilitating joint discussion and alignment. In addition, some interviewees described using the tool to reflect on ongoing or completed projects, for example, to revisit implementation processes and reconsider earlier decisions.

#### Tension Between Conceptual Depth and Practical Usability

During use, participants frequently described challenges related to the length, structure, and presentation of the accompanying materials. The guidance document was perceived as text-heavy, requiring substantial effort before actionable steps became clear. One participant stated, “I first had to work through quite a lot of text before I understood what I was actually supposed to do.” (Int_6).

A recurring issue concerned navigating between theoretical background and practical instructions. Participants reported difficulties in identifying how to move from conceptual understanding to concrete application steps. This created challenges in orienting themselves within the material, particularly under time constraints.

#### Need for Guidance and Facilitation

Participants described uncertainties regarding how to apply the tool in group-based settings, particularly with respect to structuring sessions, involving relevant stakeholders, and documenting outcomes. One participant stated, “I can see the value of the tool, but I would need more guidance on how to actually work with it in a team.” (Int_1).

Several interviewees referred to the importance of facilitation during the application, for example, by a person familiar with the tool who can guide the process and ensure that all relevant aspects are addressed. In addition, participants described difficulties in identifying concrete next steps following the use of the canvas, particularly in relation to subsequent planning or decision-making activities.

#### Flexible Use Across Implementation Phases

Participants described the NICT as applicable across different stages of implementation, with a primary focus on early phases such as project initiation and planning. In these contexts, the tool was used to gain an overview and to identify relevant factors. One participant stated, “I would definitely use it at the beginning to get an overview, but also later on to reflect on what worked and what didn’t.” (Int_8).

At the same time, some interviewees reported using the tool iteratively throughout the implementation process, for example, to reassess progress or reflect on challenges. For later stages, participants referred to the need for additional tools to support more detailed planning and management activities.

The integrated findings from the formative and summative evaluation informed the final refinement of the NICT. Revisions focused on improving clarity, usability, and practical applicability, including the provision of additional guidance, application examples, and refined instructional materials.

## Discussion

### Principal Findings

This study aimed to develop and evaluate a practice-oriented, theory-informed reflection tool to support the implementation of digital technologies in nursing. Across surveys, free-text responses, and think-aloud interviews, a consistent pattern emerged: the NICT was perceived as conceptually robust and structurally coherent, enabling systematic reflection on implementation processes. Participants particularly valued its ability to structure complexity, foster shared understanding, and support consideration of the interdependencies between care recipient-related, technology-related, user-related, and context-related factors.

Recurring critiques concerned the tool’s length, abstraction, and the need for additional implementation support during early use. These findings informed iterative refinements of the NICT, including clearer guidance and practical examples incorporated into the final version of the tool. At the same time, participants highlighted the potential value of complementary training opportunities, facilitated workshop formats, and digital versions to further support implementation in practice. Rather than indicating a fundamental trade-off between conceptual comprehensiveness and operational usability, these findings suggest that comprehensive implementation tools benefit from being embedded within broader implementation support strategies that combine structured reflection with facilitation, guidance, and context-specific learning opportunities.

This apparent tension also clarifies the role of structured reflection in implementation. The purpose of the NICT is not to reduce implementation complexity. Rather, it aims to make interacting implementation factors, implicit assumptions, competing priorities, and contextual dependencies more visible and discussable among implementation stakeholders. In this sense, the NICT supports shared understanding and context-sensitive implementation planning rather than reducing the inherent complexity of implementation. This interpretation is consistent with recent NASSS-based implementation studies, which similarly describe structured reflection as a mechanism for adaptive implementation rather than complexity reduction [24].

Beyond these practical implications, the findings reinforce the theoretical assumption that successful implementation of digital technologies in nursing requires structured reflection on interacting technical, organizational, and professional factors. This observation aligns with Greenhalgh et al [4], who conceptualize technology implementation in health care as a dynamic process shaped by interactions across multiple levels, ranging from the characteristics of the technology itself to the broader socio-organizational context.

However, implementation in nursing is a profession-specific and context-dependent process in which technologies are interpreted, negotiated, and integrated into everyday clinical practice. In line with the view of implementation as a social process of sense-making and work integration proposed by May et al [28], participants particularly valued the ability of the NICT to facilitate communication, foster shared understanding and support collective reflection among different stakeholder groups.

Importantly, this conceptual understanding was reflected in the empirical data. Participants consistently described implementation as an active, reflective process influenced by professional reasoning, situational judgment, and team-based interactions. The NICT was used to evaluate technologies and reveal implicit assumptions. It also promoted negotiation between different viewpoints and aligned implementation decisions with professional care priorities.

These findings underscore the role of nursing practice as an active mediating mechanism in implementation processes. Rather than passively adopting technologies, nurses actively shape, adapt, and sometimes resist digital innovations based on professional values, established workflows, leadership support, and care priorities [29-32].

### Relation to Prior Research

These findings align with international implementation research frameworks, such as NASSS [4], CFIR [3], as well as strategy compilations such as Expert Recommendations for Implementing Change [33], which collectively emphasize the multidimensional and context-dependent nature of implementation. Our study confirms and specifies these assumptions for nursing. Existing frameworks map complexity across domains but often underdevelop the role of professional practice. Consistent with prior research, our findings suggest that professional nursing practice should not be viewed as merely adopting change passively, but rather as a core mechanism through which digital transformation is enabled, filtered, or resisted [29,31,32].

Our findings further support understanding of implementation as a complex, adaptive process rather than a linear pathway. Organizational structures and individual engagement are interdependent: openness toward technology remains insufficient without supportive conditions, while structural resources alone do not ensure sustained use when technologies are perceived as clinically misaligned. This relational perspective was also reflected in our empirical data, where participants described implementation as shaped by the dynamic interplay of individual, organizational, and contextual factors. These observations align with prior work highlighting interactions across microlevel, mesolevel, and macrolevel [3,4,34,35].

This study reinforces a persistent challenge in implementation science: translating comprehensive implementation frameworks into practical tools for routine care [5]. Although frameworks such as NASSS provide valuable guidance for understanding implementation complexity, their application in everyday practice remains challenging [9,13]. The NICT was developed to address this gap by operationalizing implementation theory into a structured reflection tool. Our findings suggest that this translation requires balancing conceptual comprehensiveness with practical usability. Rather than reducing implementation complexity itself, structured reflection can help implementation stakeholders recognize and address complexity more systematically during implementation [36].

### Implications for Practice, Education and Research

Key implications are summarized in Textbox 1.

Textbox 1. **Implications for practice, education, and research.**

**Implications for nursing practice**

- Recognize digital implementation in nursing as a context-sensitive, profession-specific process rather than a purely technical task.
- Ensure active involvement of nursing staff across all implementation phases, from technology selection to evaluation.
- Use structured reflection tools (eg, the Nursing Implementation Complexity Tool [NICT]) within facilitated team-based reflection or implementation workshops to promote shared understanding, collaborative decision-making, and context-sensitive implementation planning.
- Support implementation through practical guidance, illustrative use cases, and clearly defined facilitation responsibilities.
- Align digital technologies with nursing workflows, time constraints, and interprofessional dependencies to avoid increasing workload.

**Implications for nursing education**

- Support the development of implementation-related competencies relevant for digitally transforming health care systems.
- Use structured tools such as the NICT to link theoretical knowledge with practical implementation challenges.
- Integrate case-based learning, guided reflection, and facilitated workshops to strengthen implementation competencies in undergraduate and continuing nursing education.

**Implications for nursing research**

- Investigate facilitated implementation approaches, including workshop-based applications of the NICT, to examine their contribution to implementation planning and team-based decision-making.
- Explore digital and AI-supported approaches (eg, dialog-based assistants) to support tool application and reduce implementation complexity.
- Conduct longitudinal and comparative studies to assess implementation outcomes, sustainability, and transferability across different nursing settings.

### Strengths and Limitations

Several limitations should be considered. First, the sample size of the summative evaluation was relatively small and based on voluntary participation, which introduces a potential self-selection bias and may limit generalizability. Second, the evaluation focused primarily on perceived usability and usefulness rather than objective implementation outcomes; therefore, no conclusions can be drawn regarding the effectiveness of the tool in improving implementation success or patient-related outcomes. Consequently, the present study should be understood as an intervention development and evaluation study rather than an effectiveness study. Third, while parts of the empirical foundation—particularly the case studies informing the conceptual development—were conducted in acute care settings, the overall evidence base (including the umbrella review and multicontext evaluation) spans a broader range of care contexts. Accordingly, the tool is not limited to acute care but may require context-specific adaptations to ensure its practical applicability across different health care settings. Finally, qualitative and quantitative data provided complementary perspectives across the development phases, with their relative contribution varying according to the specific purpose of each phase. Quantitative components were primarily descriptive and therefore complemented the more in-depth qualitative findings. An additional limitation concerns the composition of the summative evaluation sample. Among participants who reported their professional role, 14 of 21 (66.7%) were research staff or scientific personnel; similarly, 4 of the 8 interview participants were research associates in nursing science. Their familiarity with implementation concepts, innovation projects, and structured reflection may have shaped how the NICT was understood and assessed, thereby limiting the external validity of the findings for implementation stakeholders working primarily in routine care.

### Conclusions

The NICT is a theory-informed implementation reflection tool that supports nursing professionals and other implementation stakeholders in recognizing, discussing, and addressing implementation complexity during the implementation of digital technologies. Rather than reducing implementation complexity itself, the NICT structures reflection to support informed, context-sensitive implementation-related decision-making. The findings suggest that operationalizing implementation theory into practical reflection tools requires balancing conceptual comprehensiveness with usability. While the NICT was perceived as useful and well structured, participants also highlighted the need for practical guidance, examples, and facilitation, indicating that supporting reflection on implementation complexity remains a complex task in itself. Beyond its application in nursing, this study illustrates how conceptual frameworks for understanding implementation complexity can be translated into structured, practice-oriented reflection tools for digital health implementation. Future research should evaluate the application and effectiveness of the NICT across diverse health care settings and explore digital and AI-supported approaches to further support its use.

## Supplementary material

10.2196/105536 Multimedia Appendix 1 Iterative development of the Nursing Implementation Complexity Tool.

10.2196/105536 Multimedia Appendix 2 Summative survey.

10.2196/105536 Multimedia Appendix 3 Convergence coding matrix.

10.2196/105536 Multimedia Appendix 4 Nursing Implementation Complexity Tool (NICT I: guideline document (rationale, instructions, and foundations).

10.2196/105536 Multimedia Appendix 5 Nursing Implementation Complexity Tool (NICT) II: visual canvas for collaborative reflection.

10.2196/105536 Multimedia Appendix 6 Nursing Implementation Complexity Tool (NICT) III: pocket card with guiding questions and key principles.

10.2196/105536 Multimedia Appendix 7 Nursing Implementation Complexity Tool (NICT) application—example use case.

## Abbreviations

CFIR: Consolidated Framework for Implementation Research
DSR: Design Science Research
FT: free-text item
GRAMMS: Good Reporting of a Mixed Methods Study
GUIDED: Guidance for Reporting Intervention Development Studies in Health Research
NASSS: Nonadoption, Abandonment, Scale-Up, Spread, and Sustainability
NICT: Nursing Implementation Complexity Tool
SRQR: Standards for Reporting Qualitative Research
