# Supplementary material for: The Nursing Implementation Complexity Tool for Digital Technology Implementation in Nursing: Design Science Research Study
Source: J Med Internet Res. 2026 Sep 30;28:e105536. doi: 10.2196/105536 (PMC13626400; doi:10.2196/105536)
Supplement: Supplementary file 1 [file jmir-v28-e105536-s001.pdf]

Iterative development of the Nursing Implementation Complexity Tool (NICT) across the four Design Science Research phases.

DSR phase*	Evidence source	Purpose and contribution	Contribution to the iterative development of the NICT
**Phase 1 – Conceptual foundation**	Umbrella review	Synthesized evidence on barriers, facilitators, and implementation determinants influencing the implementation and adoption of digital nursing technologies across the NASSS domains.	Established the theoretical foundation of the NICT by identifying implementation determinants relevant to nursing practice.
	Three mixed-methods case studies	Contributed empirical evidence on implementation experiences, adoption dynamics, contextual constraints, and user perspectives from real-world implementation projects.	Refined the understanding of nursing-specific implementation challenges and contextual requirements, ensuring that empirical implementation experiences complemented the theoretical evidence.
	NASSS-CAT-D	Provided a German adaptation of the NASSS Complexity Assessment Tool as an implementation support tool and an initial operationalization of implementation complexity.	Informed the initial conceptual organization of the NICT and supported the translation of implementation theory into a practical reflection format.
	**Integrated synthesis**	Findings from all three sources were systematically mapped onto the NASSS framework and iteratively discussed within the research team.	Resulted in four reflection domains (care recipients, health professionals, technology, organizational context) with ethical and legal considerations as cross-cutting dimensions, forming the conceptual foundation of the NICT.

**Phase 2 – Tool development**	Findings from Phase 1	Implementation determinants and contextual requirements were translated into practical reflection domains, guiding questions, application examples, and visual representations.	Development of the first NICT prototype consisting of a narrative guideline, visual canvas, and pocket card.
	Structured working sessions, interdisciplinary research meetings, and iterative feedback loops	Supported continuous refinement of the content, wording, structure, and usability of the prototype through collaborative discussion.	Progressive refinement of the prototype before expert review.
	Expert review (n = 9)	Stakeholders from nursing management, advanced nursing practice, clinical ethics, information technology, and data protection evaluated completeness, clarity, relevance, and practical applicability.	Simplified language, improved guidance, enhanced visual presentation, and additional practical examples resulted in **Version 1** of the NICT.
**Phase 3 – Testing**	Participatory workshops (conference participants, prospective nurse managers, nursing students)	Evaluated usability, comprehensibility, and perceived practical relevance across different educational and professional contexts.	Confirmed the conceptual structure of the NICT while identifying practical requirements for successful workshop implementation.
	Canvas templates, participant feedback, facilitator field notes	Generated evidence regarding collaborative reflection processes, usability challenges, contextual requirements, and facilitation needs.	Led to refinements of workshop procedures, implementation scenarios, instructional guidance, and facilitation recommendations without substantial changes to the core structure of the NICT.
**Phase 4 – Summative evaluation**	Standardized online survey	Quantitatively evaluated usability, clarity, perceived usefulness, and transferability of the NICT across nursing contexts.	Confirmed the overall structure of the NICT and identified areas requiring further refinement.
	Survey free-text responses	Provided detailed feedback regarding practical application, transferability,	Informed revisions of guidance materials and highlighted the need for supporting

		implementation support, and additional resource needs.	examples, digital formats, and training materials.
	Think-aloud interviews	Generated in-depth insights into user interaction, cognitive processes, navigation, and practical application of the NICT.	Supported the final refinement of guidance materials, application examples, and implementation recommendations.
	**Integrated synthesis**	Quantitative and qualitative findings were considered together during interpretation to identify converging evidence and remaining improvement needs (**see Multimedia Appendix 2**).	Final version of the NICT emphasizing clarity, usability, practical guidance, and implementation support.

* The table summarizes the empirical evidence informing each phase of the development process and illustrates how theoretical knowledge, empirical findings, and iterative evaluation contributed to successive refinements of the NICT.
