# Supplementary material for: The Nursing Implementation Complexity Tool for Digital Technology Implementation in Nursing: Design Science Research Study
Source: J Med Internet Res. 2026 Sep 30;28:e105536. doi: 10.2196/105536 (PMC13626400; doi:10.2196/105536)
Supplement: Supplementary file 2 [file jmir-v28-e105536-s002.pdf]

LimeSurvey

Herzlich willkommen und vielen Dank für Ihr Interesse an der Online-Befragung zur Evaluation der Handlungsempfehlung zur Reflexion und Bewertung von digitalen Technologien in der Pflege.

Ziel dieser Befragung ist es, die Verständlichkeit, Anwendbarkeit und Praxistauglichkeit der Handlungsempfehlung – einschließlich Canvas und Pocketcard – aus Ihrer Perspektive zu erfassen. Dabei interessiert uns insbesondere, inwieweit die Handlungsempfehlung Sie bei der Reflexion und Planung von Technologieimplementierungen in der Pflege unterstützt und ob sie sich auch für andere Versorgungskontexte (z. B. Langzeit- oder Rehabilitationspflege) eignet.

Die Ergebnisse dieser Befragung sollen zur Weiterentwicklung und Optimierung der Handlungsempfehlung beitragen und die Grundlage für weitere Evaluationen bilden.

Einige der Fragen sind als Pflichtfelder gekennzeichnet. Falls Ihnen eine Einschätzung schwerfällt, steht bei den meisten Fragen die Antwortoption „kann ich nicht beurteilen“ zur Verfügung. Bitte beantworten Sie die Fragen auf Basis Ihrer persönlichen Einschätzung und Erfahrung.

Die Befragung dauert etwa 10 bis 15 Minuten. Ihre Teilnahme ist freiwillig und anonym. Alle Angaben werden vertraulich behandelt und ausschließlich für wissenschaftliche Zwecke ausgewertet.

Vielen Dank für Ihre Unterstützung!

Teil A: Ihre Forschungsnummer

Zunächst bitten wir Sie, sich eine eigene 4-stellige Forschungsnummer zu erstellen. Dadurch können wir im Falle des Gebrauchs Ihrer Rechte (z.B. bei Widerruf) eine korrekte Zuordnung zu den Forschungsdaten gewährleisten.

Die entsprechenden Angaben können Sie in das jeweilige Freitextfeld eintragen (bei Buchstaben können Sie gerne Kleinbuchstaben verwenden). Beispiel: 1. Anfangsbuchstabe des Vornamens der Mutter -> **a**nna 2. Letzte Ziffer des eigenen Geburtsjahres -> 197**4** 3. Zweiter Buchstabe des Geburtsmonates der Mutter -> f**e**bruar 4. Anfangsbuchstabe des eigenen Geburtsortes -> **h**eidelberg Die Forschungsnummer würde in diesem Fall dann so aussehen: a4eh

A1. 1. Anfangsbuchstabe des Vornamens der Mutter

LimeSurvey

A2. **2. Letzte Ziffer des eigenen Geburtsjahres**

A3. **3. Zweiter Buchstabe des Geburtsmonates der Mutter**

A4. **4. Anfangsbuchstabe des eigenen Geburtsortes**

Teil B: Teil A: Bewertung der Handlungsempfehlung

B1.

	stimme gar nicht zu	stimme nicht zu	stimme weniger zu	stimme eher zu	stimme voll zu	kann ich nicht beurteilen
1. Insgesamt denke ich, dass die Handlungsempfehlung hilfreich zur Implementierung von Technologien in der Pflege ist.	☐	☐	☐	☐	☐	☐
2. Hätte es diese Handlungsempfehlung bereits früher in meiner Einrichtung bzw. meinem Projekt gegeben, hätte ich sie wahrscheinlich genutzt.	☐	☐	☐	☐	☐	☐
3. Die Handlungsempfehlung ist sprachlich leicht verständlich formuliert.	☐	☐	☐	☐	☐	☐
4. Die Handlungsempfehlung lässt sich einfach und effizient anwenden.	☐	☐	☐	☐	☐	☐

Teil C: Teil A: Bewertung der Handlungsempfehlung

C1.

	stimme gar nicht zu	stimme nicht zu	stimme weniger zu	stimme eher zu	stimme voll zu	kann ich nicht beurteilen
5. Die Inhalte der Handlungsempfehlung (inkl. Pocketcard und Canvas) sind logisch aufgebaut.	☐	☐	☐	☐	☐	☐
6. Die Handlungsempfehlung ermöglicht mir, ein umfassendes Bild meines Implementierungsvorhabens zu gewinnen.	☐	☐	☐	☐	☐	☐
7. Die Handlungsempfehlung fördert die Reflexion über relevante Erfolgsfaktoren und Stolpersteine bei Technologieprojekten.	☐	☐	☐	☐	☐	☐

LimeSurvey

	stimme gar nicht zu	stimme nicht zu	stimme weniger zu	stimme eher zu	stimme voll zu	kann ich nicht beurteilen
8. Das Canvas unterstützt mich dabei, komplexe Implementierungsprozesse zu strukturieren und zu visualisieren.	☐	☐	☐	☐	☐	☐

Teil D: Teil A: Bewertung der Handlungsempfehlung

D1.

	stimme gar nicht zu	stimme nicht zu	stimme weniger zu	stimme eher zu	stimme voll zu	kann ich nicht beurteilen
9. Die Pocketcard bietet eine gute Ergänzung zum Canvas und erleichtert den Einstieg in die Reflexion.	☐	☐	☐	☐	☐	☐
10. Die Handlungsempfehlung eignet sich grundsätzlich auch für andere pflegerische Settings (z. B. Langzeitpflege, ambulante Pflege, Reha).	☐	☐	☐	☐	☐	☐
11. Ich kann mir vorstellen, die Handlungsempfehlung in Schulungen oder Lehre einzusetzen.	☐	☐	☐	☐	☐	☐
12. Ich halte eine digitale Version der Handlungsempfehlung (Canvas/Pocketcard) für sinnvoll.	☐	☐	☐	☐	☐	☐

Teil E: Teil B: Freitextfragen

E1. **1. Welche Elemente der Handlungsempfehlung (Text, Canvas, Pocketcard) waren für Sie besonders hilfreich – und warum?**

E2. **2. Gab es Stellen, die Sie als unverständlich, zu komplex oder nicht praxisnah empfanden?**

E3. **3. In welchen Situationen oder Phasen (z. B. Projektstart, Planung, Evaluation) würden Sie die Handlungsempfehlung einsetzen?**

E4. **4. Wie gut halten Sie die Handlungsempfehlung für andere Pflegekontexte (z. B. Langzeitpflege, Reha, ambulante Pflege) geeignet?**

Teil F: Teil B: Freitextfragen

F1. **5. Was müsste für den Einsatz in anderen Settings ggf. angepasst werden?**

F2. **6. Welche zusätzlichen Materialien oder Unterstützungsangebote (z. B. Beispiel-Canvas, Schulung, digitale Version) würden die Nutzung erleichtern?**

LimeSurvey

F3. **7. Haben Sie noch weitere Anmerkungen, Wünsche oder Verbesserungsvorschläge?**

Teil G: Optional: Angaben zur Person

G1. **1. In welcher Funktion / Rolle sind Sie tätig?**

G2. **2. Berufserfahrung in Jahren**

G3. **3. Erfahrung mit Technologieimplementierung:**

- keine ☐
- gering ☐
- mittel ☐
- hoch ☐

G4. **4. Ihr primäres pflegerisches Setting:**

G5. **5. Sind Sie damit einverstanden, dass wir Sie ggf. für eine zusätzliche vertiefende Think-Aloud-Erhebung (ca. 20–30 Minuten, online) kontaktieren dürfen? Wenn ja, können Sie hier unverbindlich eine E-Mail-Adresse angeben, unter der wir Sie erreichen können.**

Vielen Dank für Ihre Teilnahme an dieser Umfrage!

Ihre Umfrage-ID lautet: {SID}

Bitte drucken Sie Ihre Antworten der Befragung aus, damit Sie u.a. im Falle eines späteren Widerrufs Ihre persönliche Forschungsnummer griffbereit haben und unter Angabe dieser Nummer die Löschung Ihrer Daten veranlassen können.
