# Supplementary material for: The Nursing Implementation Complexity Tool for Digital Technology Implementation in Nursing: Design Science Research Study
Source: J Med Internet Res. 2026 Sep 30;28:e105536. doi: 10.2196/105536 (PMC13626400; doi:10.2196/105536)
Supplement: Supplementary file 3 [file jmir-v28-e105536-s003.pdf]

Multimedia Appendix 2 - Convergence Coding Matrix: Integration of quantitative and qualitative findings informing the final refinement of the Nursing Implementation Complexity Tool (NICT)

Theme	Quantitative survey	Survey free-text responses	Think-aloud interviews	Convergence	Influence on the iterative refinement of the NICT
**1. Overall usability and perceived usefulness**	Helpful for implementation: 69.0%, mean 3.93 (SD 1.14); Would have used earlier: 66.7%, mean 3.56 (SD 1.28)	Overall concept described as useful, practice-oriented, and relevant for implementation planning.	Participants consistently described the NICT as valuable for structuring implementation processes and interdisciplinary discussion.	Agreement	Overall concept retained.
**2. Logical structure and organization**	Logical structure: 92.3%, mean 4.27 (SD 0.69); Comprehensive overview: 76.9%, mean 4.00 (SD 0.98)	Canvas and pocket card perceived as providing structure and orientation.	Participants appreciated the systematic organization and visual representation of implementation complexity.	Agreement	Core structure retained.
**3. Ease of use and clarity**	Easy to understand: 67.9%, mean 3.93 (SD 1.08); Easy to apply: 64.0%, mean 3.64 (SD 1.05)	Requests for simpler language, shorter guidance, clearer explanations, and improved relationship between guideline, canvas and pocket card.	Initial orientation difficulties; uncertainty regarding navigation between theoretical background and practical application.	Partial agreement	Guidance revised, wording simplified, interaction between tool components clarified.
**4. Structured reflection on implementation complexity**	Supports reflection: 92.3%, mean 4.38 (SD 0.75); Canvas structures complexity: 73.1%, mean 3.92 (SD 0.97)	Participants reported that the NICT supported identification of barriers, facilitators and relevant stakeholder perspectives.	Participants described becoming aware of aspects that might otherwise have been overlooked and jointly reflecting on implementation complexity.	Agreement	Reflection domains and guiding questions retained.
**5. Practical implementation support**	Helpful for implementation: 69.0%; Easy to apply: 64.0%	Frequent requests for example canvases, case studies, practical guidance, and implementation examples.	Participants requested clearer guidance on how to use the NICT in real implementation projects and how to proceed after completing the reflection process.	Agreement	Additional examples, practical guidance and supporting materials incorporated.

**6. Facilitation and workshop application**	No dedicated quantitative item.	Participants suggested workshops, short training formats, instructional videos and collaborative learning formats.	Participants emphasized the importance of facilitation, sufficient time, smaller group sizes and detailed implementation scenarios.	Silence (quantitative); Agreement (qualitative)	Recommendations for facilitated workshops, revised workshop guidance and improved scenarios.
**7. Digital implementation support**	Digital version useful: 83.3%, mean 4.17 (SD 0.96)	Requests for digital versions, videos and collaborative tools.	Participants considered digital support a valuable extension rather than a replacement of the NICT.	Agreement	Digital version identified as future development priority.
**8. Transferability across settings**	Suitable for other settings: 73.1%, mean 4.00 (SD 1.06); Suitable for teaching: 68.2%, mean 3.73 (SD 1.18)	Participants considered the NICT transferable but recommended setting-specific examples and adaptations.	Participants described broad applicability while emphasizing context-specific adaptation and iterative use across implementation phases.	Agreement	Core framework retained; setting-specific supporting materials recommended.

*Convergence categories adapted from Farmer et al. (2006): **Agreement**, **Partial agreement**, **Silence**, and **Dissonance**. No substantial dissonance between quantitative and qualitative findings was identified in the present study.
