# Supplementary material for: The Nursing Implementation Complexity Tool for Digital Technology Implementation in Nursing: Design Science Research Study
Source: J Med Internet Res. 2026 Sep 30;28:e105536. doi: 10.2196/105536 (PMC13626400; doi:10.2196/105536)
Supplement: Supplementary file 4 [file jmir-v28-e105536-s005.pdf]

NICT Canvas

Reflection domains for the implementation of digital technologies in nursing

Aim

Healthcare professionals

Care recipients

Technology

Organisational context

Ethical and legal aspects

Cross-cutting theme relevant across all reflection domains: data protection and privacy, autonomy and consent, justice and equity, non-maleficence and safety, transparency, accountability, and applicable legal and regulatory requirements. **What to document:** Relevant ethical and legal considerations, risks, responsibilities, and requirements that should be taken into account across the reflection domains.

This canvas supports the reflection on and evaluation of digital technologies in nursing. Some factors in your project may not be clearly assignable to a single domain. In such cases you can assign them to several domains or consider them from the perspective that is most relevant to you. At this stage it is important to emphasize that identifying as many relevant factors as possible matters more than strict categorisation. Please ensure that ethical and legal aspects are considered and documented in line with the chosen perspective.
