# Supplementary material for: The Nursing Implementation Complexity Tool for Digital Technology Implementation in Nursing: Design Science Research Study
Source: J Med Internet Res. 2026 Sep 30;28:e105536. doi: 10.2196/105536 (PMC13626400; doi:10.2196/105536)
Supplement: Supplementary file 5 [file jmir-v28-e105536-s006.pdf]

NICT Pocket card

Reflection domains for the implementation of digital technologies in nursing

FRONT

Aim
The intended purpose and the desired outcomes.

Healthcare professionals

Professionals who interact with the technology and integrate it into care processes.

What to document: Factors that influence the acceptance and use of the technology in everyday care.

Guiding questions

- How can the technology be integrated into care workflows? → Prior knowledge, professional identity and role perception
- To what extent is the technology accepted by nursing professionals and patients? → Sense of safety, curiosity, motivation, transparency of the implementation process
- How can the (technical) competence of the nursing staff in relation to the technology be assessed? → Prior experience, technological affinity, training needs
- How likely is it that the expected added value for end users can actually be realised?
- Which ethical and legal aspects need to be considered? → For further details see the back

Care recipients

People receiving care whose needs, experiences and outcomes are influenced by the technology.

What to document: Characteristics, needs and experiences of care recipients that influence the technology or may be influenced by it.

Guiding questions

- Which characteristics and attributes can be used to describe the target group? → Commonalities and heterogeneity
- Which groups of care recipients are suitable for the intended application scenario?
- Which decision-support or assessment instruments facilitate the identification of the target group?
- How likely is it that the expected benefits for care recipients can actually be achieved?
- Which ethical and legal aspects need to be considered? → For further details see the back

Technology

The digital system itself, including functions, usability, reliability and integration into existing infrastructures.

What to document: Characteristics of the technology that influence use, integration and performance in care practice.

Guiding questions

- How practicable is the technology? → Usability and potential applications
- How reliable is the technology? → Functionality, accuracy and complementary components
- What is known about the support provided by the manufacturer?
- What added value does the manufacturer claim for the technology?
- How is the additional value stated by the manufacturer evidenced or substantiated?
- Which ethical and legal aspects need to be considered? → For further details see the back

Organisational context

The organisational, structural and regulatory environment in which the technology is implemented and used.

What to document: Contextual conditions at organisational and system level that influence implementation and sustainability.

Guiding questions

- How receptive is the organisation to innovation?
- Which resources are required and which are available? → Staff, financial resources, physical space, knowledge management
- Which (technical) infrastructure is required for implementation? → Wi-Fi, interfaces, integration into the electronic patient record system
- Which stakeholders are relevant for the implementation? → e.g. infection control, occupational safety and health, biomedical engineering
- How likely is it that the expected added value can actually be achieved in this setting?
- Which ethical and legal aspects need to be considered? → For further details see the back

This pocket card supports the reflection on and assessment of digital technologies in nursing by taking key implementation aspects into account. Every implementation situation brings its own context-specific challenges, which is why this pocket card does not claim to be exhaustive. Rather, it is intended to support a structured, multidimensional assessment of the context in which implementation takes place.

NICT Pocket card

Reflection domains for the implementation of digital technologies in nursing

BACK

Aim
The intended purpose and the desired outcomes.

Ethical aspects

Guiding principle

Step back from everyday practice and routines and reflect on the values and goals that are fundamental to high-quality care.

The following aspects can support your reflection:

- Consider the different domains in relation to values and goals (e.g. care recipients, users, organisational setting).
- Weigh up the implications of technological possibilities: is the technology designed in a way that restricts care recipients, or does it support and empower them? Are there alternative technologies or modes of use?
- Identify necessary adaptations in the environment in which the technology is used (e.g. changes to existing routines, including whether users are able to respond to technological requirements).
- Careful selection of the care recipients for whom the technology is suitable (e.g. case-by-case assessment and situational decision-making).
- Reflect on the effects of the technology on the (professional) self-image of care recipients and staff (e.g. ensuring flexibility in care; experiencing oneself as an autonomous individual).

The following aspects should be examined critically:

- Core values of care may be jeopardised or only partly preserved (e.g. privacy, autonomy, safety, care, benefit and relational aspects of care).
- Perceived benefit for the organisation or the staff may be prematurely assumed to be a benefit for care recipients (or vice versa), without critical examination.
- Needs, concerns or tensions expressed by care recipients are not taken seriously (e.g. regarding monitoring), which may lead to paternalistic practices (restriction of autonomy), particularly for people with cognitive impairments.
- Needs of the staff are not adequately taken into account (e.g. regarding monitoring, quality of care or increased workload).

Legal aspects

Personal rights:

Informed consent

→ Does the person have the capacity to give informed consent?
→ Is support for decision-making required?
→ Is consent obtained from authorised representatives (e.g. family members, legal representatives, court-appointed guardians) where applicable?
→ Appropriate form and documentation of consent in accordance with applicable regulations.

Dignity

→ Right to privacy and to withdraw consent
→ Avoidance of humiliation or degrading treatment (e.g. violations of autonomy, paternalistic practices)

Data protection and privacy:

→ Which types of data are collected, stored and processed?
→ Is the collection and storage of the data necessary and proportionate?
→ Is there valid informed consent or another appropriate legal basis for the data processing?
→ Is the purpose of the data use clearly defined and adhered to (purpose limitation)?
→ How is access to data regulated and protected (e.g. through authorisation, security measures)?

Liability and accountability:

→ Is appropriate qualification for the use of the technology ensured?
  → professional competencies (through training and instruction)
  → formal qualification (where required by legal frameworks)
→ Which internal guidelines or standards govern the use of the technology?
→ Are procedures in place for handling adverse events or harm?
→ Is the use of the technology covered by professional liability or institutional insurance?

Professional and regulatory aspects:

→ Does the use of the technology fall under:
  → the medical domain (e.g. diagnosis, treatment)?
  → the nursing or care domain (e.g. professional scope of practice, care processes)?
  → interprofessional collaboration and shared responsibility?
→ Where applicable: delegation of the use of the technology and supervision in accordance with professional and regulatory standards.

This pocket card supports the reflection on and assessment of digital technologies in nursing by taking key implementation aspects into account. Every implementation situation brings its own context-specific challenges, which is why this pocket card does not claim to be exhaustive. Rather, it is intended to support a structured, multidimensional assessment of the context in which implementation takes place.
