# Supplementary material for: The Nursing Implementation Complexity Tool for Digital Technology Implementation in Nursing: Design Science Research Study
Source: J Med Internet Res. 2026 Sep 30;28:e105536. doi: 10.2196/105536 (PMC13626400; doi:10.2196/105536)
Supplement: Supplementary file 6 [file jmir-v28-e105536-s004.docx]

**Nursing Implementation**

**Complexity Tool (NICT)**

Guideline

Structured reflection for the implementation of digital technologies in nursing

|  | **PURPOSE** The NICT helps teams identify, discuss, assess, and document context-specific implementation factors before and during technology-supported change. |
| --- | --- |

**The NICT consists of three complementary components**

| **Canvas**  Structures and documents the reflection. | **Pocket card**  Provides guiding questions for each domain. | **Guideline**  Explains the rationale and practical application. |
| --- | --- | --- |

*Use this guideline for preparation, facilitation, and follow-up. The canvas and pocket card remain the core working tools.*

# 2. Purpose and scope

The NICT supports systematic reflection on and assessment of digital technology implementation in nursing. It is intended for nursing professionals, leaders, educators, implementation facilitators, and other stakeholders involved in planning, introducing, adapting, or evaluating technologies.

The tool is setting-independent within nursing and can be adapted for acute care, long-term care, outpatient care, rehabilitation, and other nursing contexts. It does not provide a universal implementation pathway or replace technology-specific, organizational, ethical, legal, regulatory, or data-protection review.

|  | **GUIDING PRINCIPLE** Every implementation situation is context-specific. Use the NICT to make relevant assumptions, risks, responsibilities, and trade-offs explicit rather than to generate a single score or pass/fail decision. |
| --- | --- |

# 2. Applying the NICT

The process can begin with a concrete need or problem in nursing practice, or with an existing or proposed technology whose suitability and contextual fit need to be assessed.

| **1** | **Define the starting point and aim**  Describe the need, problem, or technology and formulate what the implementation is intended to achieve. |
| --- | --- |

| **2** | **Structure the reflection**  Use the canvas and pocket-card questions to consider relevant factors across all reflection domains. |
| --- | --- |

| **3** | **Discuss and assess collectively**  Bring together different perspectives, identify tensions and dependencies, and agree on conclusions and next actions. |
| --- | --- |

| **4** | **Document and revisit**  Record key considerations, open questions, responsibilities, and decisions. Repeat the reflection as the context or technology changes. |
| --- | --- |

|  | **ITERATIVE USE** The NICT can be used at project initiation, during selection and procurement, before piloting, during implementation, and when reviewing adaptation, scale-up, or sustainability. |
| --- | --- |

# 3. Involving stakeholders

Effective reflection requires the early involvement of people who are affected by, use, enable, govern, or make decisions about the technology. Participation should be adapted to the implementation context.

| **Practice and leadership**  Nursing professionals; nursing leadership; advanced practice and education roles; other professional groups as relevant | **Care recipients**  Patients, residents, clients, relatives, representatives, and advocacy perspectives as appropriate |
| --- | --- |
| **Technical and organizational support**  IT; medical technology; information security; procurement; quality management; human resources | **Governance and assurance**  Data protection; legal services; clinical ethics; occupational safety; regulatory or works-council representation |

|  | **FACILITATION** A person familiar with the NICT can guide the process, ensure that all domains are addressed, make differing perspectives visible, and support documentation, especially during initial use. |
| --- | --- |

# 4. Reflection domains

The four domains capture central and interrelated aspects of digital technology implementation in nursing. Relevant factors may relate to more than one domain; assign them where they are most useful for reflection and cross-reference dependencies where needed.

| **Healthcare professionals**  **Focus** Needs, roles, competencies, attitudes, workload, professional autonomy, collaboration, training, and support.  **What to document** Perspectives, anticipated effects, competency and support needs, responsibilities, and unresolved concerns. | **Care recipients**  **Focus** Needs and characteristics, safety, accessibility, autonomy, participation, experience of care, and potential benefits or burdens.  **What to document** Relevant needs, expected effects, risks, involvement, safeguards, and accessibility requirements. |
| --- | --- |
| **Technology**  **Focus** Functionality, usability, reliability, evidence, interoperability, data flows, maintenance, technical support, and fit with workflows.  **What to document** Required functions, limitations, dependencies, integration requirements, testing needs, and support arrangements. | **Organizational context**  **Focus** Structures, workflows, resources, leadership, implementation capacity, communication, procurement, financing, and local conditions.  **What to document** Contextual enablers and barriers, resources, responsibilities, process changes, decisions, and implementation conditions. |

| **Ethical and legal aspects** | **Cross-cutting theme** Data protection and privacy, autonomy and consent, justice and equity, non-maleficence and safety, transparency, accountability, and applicable legal and regulatory requirements.  **What to document** Relevant ethical and legal considerations, risks, responsibilities, safeguards, and requirements to be taken into account across the reflection domains. |
| --- | --- |

# 5. Using the tools together

| **Canvas**  Use it to visualize, structure, and document the reflection. Begin by recording the implementation aim. | | **Pocket card**  Use the guiding questions selectively. Questions are prompts, not a mandatory checklist or exhaustive assessment. | **Guideline**  Use it to prepare and facilitate the process, clarify concepts, and support follow-up and repeated reflection. |
| --- | --- | --- | --- |
|  | **DOCUMENTATION** Record the most decision-relevant points. Distinguish between current knowledge, assumptions, open questions, agreed actions, responsible persons, and issues that require specialist review. | | |

# 6. Background and rationale

|  | **OPTIONAL BACKGROUND** This section provides conceptual context. It is not required for the practical application of the canvas and pocket card. |
| --- | --- |

## Why structured reflection is needed

Digital technologies are increasingly introduced into nursing to support quality, safety, communication, coordination, and efficiency. Yet successful integration into routine care cannot be assumed. Implementation is shaped by an interplay of clinical, organizational, professional, technical, ethical, and legal factors. Technologies that appear promising may remain unused, be adapted in unintended ways, or fail to deliver sustainable value when these factors are not addressed.

## A context-dependent process

The NICT is based on the view that implementation is not merely a technical or administrative task. Technologies enter existing care relationships, professional routines, workflows, infrastructures, and organizational conditions. Their effects depend on how well they fit the needs of care recipients, the perspectives and competencies of healthcare professionals, the properties of the technology, and the organizational context in which it is used.

## From implementation knowledge to a practical tool

The NICT was informed by empirical implementation experience, scientific evidence on factors influencing the implementation and adoption of digital technologies in nursing, and the NASSS framework and NASSS-CAT. Across these sources, implementation is supported when the technology addresses a meaningful need, expected benefits and trade-offs are transparent, users are involved and prepared, responsibilities and resources are clarified, and the technology fits local systems and workflows.

Existing frameworks offer conceptual depth but can be difficult to apply directly in everyday nursing contexts. The NICT translates complexity-informed implementation knowledge into a compact reflection approach. It emphasizes orientation and critical discussion rather than prescription, and is intended to make implicit assumptions, dependencies, tensions, and unresolved questions visible.

## Interpretation and limitations

The NICT does not predict implementation success, generate a validated complexity score, or replace formal evaluation. Its domains and questions are deliberately broad and must be interpreted in relation to the specific technology, users, care setting, implementation phase, and intended outcomes. Conclusions should therefore be documented transparently and revisited when conditions change.

|  | **CORE PROPOSITION** Sustainable technology implementation in nursing requires more than innovation readiness or technical feasibility. It requires shared reflection on relevance, fit, consequences, responsibilities, and context. |
| --- | --- |

# 7. Further reading

The following sources provide background on the development of the NICT, complexity-informed technology implementation, and ethical and legal reflection.

## Frameworks for technology implementation

Greenhalgh T, Wherton J, Papoutsi C, et al. Beyond Adoption: A New Framework for Theorizing and Evaluating Nonadoption, Abandonment, and Challenges to the Scale-Up, Spread, and Sustainability of Health and Care Technologies. Journal of Medical Internet Research. 2017;19(11):e367. [doi:10.2196/jmir.8775](https://doi.org/10.2196/jmir.8775)

Greenhalgh T, Maylor H, Shaw S, et al. The NASSS-CAT Tools for Understanding, Guiding, Monitoring, and Researching Technology Implementation Projects in Health and Social Care: Protocol for an Evaluation Study in Real-World Settings. Journal of Medical Internet Research. 2020;22(5):e16861. [doi:10.2196/16861](https://doi.org/10.2196/16861)

Damschroder LJ, Reardon CM, Widerquist MAO, Lowery J. The updated Consolidated Framework for Implementation Research based on user feedback. Implementation Science. 2022;17:75. [doi:10.1186/s13012-022-01245-0](https://doi.org/10.1186/s13012-022-01245-0)

May CR, Mair F, Finch T, et al. Development of a theory of implementation and integration: Normalization Process Theory. Implementation Science. 2009;4:29. [doi:10.1186/1748-5908-4-29](https://doi.org/10.1186/1748-5908-4-29)

Yusof MM, Kuljis J, Papazafeiropoulou A, Stergioulas LK. An evaluation framework for Health Information Systems: human, organization and technology-fit factors (HOT-fit). International Journal of Medical Informatics. 2008;77(6):386-398. [doi:10.1016/j.ijmedinf.2007.08.011](https://doi.org/10.1016/j.ijmedinf.2007.08.011)

van Gemert-Pijnen JEWC, Nijland N, van Limburg M, et al. A holistic framework to improve the uptake and impact of eHealth technologies. Journal of Medical Internet Research. 2011;13(4). [doi:10.2196/jmir.1672](https://doi.org/10.2196/jmir.1672)

## Ethical and legal reflection

International Council of Nurses. The ICN Code of Ethics for Nurses. Geneva: International Council of Nurses; 2021. Available from: <https://www.icn.ch/resources/publications-and-reports/icn-code-ethics-nurses>

World Health Organization. Ethics and Governance of Artificial Intelligence for Health: WHO Guidance. Geneva: World Health Organization; 2021. Available from: <https://www.who.int/publications/i/item/9789240029200>

Ramvi E, Lavik MH, Nygaard MR. Ethics of care in technology-mediated healthcare practices: a scoping review. Scand J Caring Sci. 2023;37(4):1162-1175. [doi:10.1111/scs.13186](https://onlinelibrary.wiley.com/doi/10.1111/scs.13186)

Korhonen ES, Nordman T, Eriksson K. Technology and its ethics in nursing and caring journals: an integrative literature review. Nurs Ethics. 2015;22(5):561-576. [doi:10.1177/0969733014549881](https://journals.sagepub.com/doi/10.1177/0969733014549881?url_ver=Z39.88-2003&rfr_id=ori:rid:crossref.org&rfr_dat=cr_pub%20%200pubmed)

|  | **IMPORTANT** These sources provide orientation. Applicable local, national, and European requirements, organizational policies, professional standards, and specialist advice must be considered for each implementation situation. |
| --- | --- |
