# Supplementary material for: The Nursing Implementation Complexity Tool for Digital Technology Implementation in Nursing: Design Science Research Study
Source: J Med Internet Res. 2026 Sep 30;28:e105536. doi: 10.2196/105536 (PMC13626400; doi:10.2196/105536)
Supplement: Supplementary file 7 [file jmir-v28-e105536-s007.docx]

**Nursing Implementation**

**Complexity Tool (NICT)**

Example Use Case

In-bed motion monitoring on a geriatric ward

|  | **PURPOSE** The following example shows how the NICT can be used to structure reflection and support discussion in a real-world care context. For illustrative purposes, the domains are presented in narrative form, while the original canvas format is particularly suited for collaborative use in group settings. |
| --- | --- |

# 1. Use case and reflection aim

A geriatric ward in a hospital considers implementing an in-bed motion monitoring system to support patient observation and nursing care. Patients in this setting often present with frailty, multimorbidity, mobility limitations, and an increased risk of falls, delirium, and pressure ulcers. The system continuously captures patient movement (e.g., repositioning, restlessness, or attempts to leave the bed) and provides real-time information to nursing staff.

The technology is intended to contribute to fall prevention and to support pressure ulcer prevention by indicating whether patients reposition themselves sufficiently during rest periods. This may enable a shift from standardized repositioning routines toward a more individualized, needs-based approach, for example by reducing unnecessary nighttime repositioning when sufficient movement is detected.

At the same time, the implementation raises questions regarding its fit within existing workflows, its impact on professional decision-making, and its implications for patient experience, autonomy, and privacy.

The NICT is used to support a structured reflection on these aspects and to assess the suitability of the technology within the specific care context.

|  | **REFLECTION AIM** Assess whether, for whom, under which conditions, and at what stage of care the system could provide meaningful support without creating disproportionate burdens, risks, or restrictions. |
| --- | --- |

# 2. Healthcare professionals

**Healthcare professionals**

**Potential contribution** Real-time information on in-bed movement, movement toward the bed edge, or bed-exit events may increase situational awareness, reduce uncertainty, and support prioritization and individualized clinical reasoning.

**Questions and risks** Staff must interpret movement information and alerts in relation to the individual patient situation and decide whether and how to respond. Poorly configured or frequent alerts may increase cognitive load, interrupt work, and reduce trust in the system. New competencies, responsibilities, and escalation routines may be required.

**What to document** User groups and roles; expected effects on workload, observation practices, and clinical decisions; training and support needs; responsibilities for interpreting information and responding to alerts; concerns, acceptance, and feedback mechanisms.

# 3. Care recipients

**Care recipients**

**Potential contribution** Earlier recognition of relevant movement patterns, movement toward the bed edge, or bed-exit events may support timely assistance. More differentiated movement information may also help avoid unnecessary routine checks or disturbances when clinically appropriate.

**Questions and risks** Continuous monitoring of movement may be experienced as intrusive. Effects on autonomy, dignity, privacy, sleep, and perceived control may differ between individuals, particularly when cognitive capacity, orientation, or communication needs fluctuate.

**What to document** Individual needs, preferences, and reasons for monitoring; anticipated benefits and burdens; accessibility and communication requirements; involvement of patients or representatives; and criteria for individualized initiation, adaptation, and discontinuation of monitoring.

# 4. Technology

**Technology**

**Potential contribution** The system can continuously capture predefined movement-related information, such as changes in in-bed activity, movement toward the bed edge, or bed-exit events, and provide corresponding information or alerts to healthcare professionals.

**Questions and risks** Clinical usefulness depends on whether relevant movement events are detected reliably and whether the information provided is sufficiently specific and actionable. False, delayed, missed, or non-specific alerts may lead to inappropriate responses or alert fatigue. Usability, configurability, system availability, maintenance, cybersecurity, and integration with existing technical systems must also be considered.

**What to document** Movement events and functions to be detected; intended use and limitations; performance and relevant error patterns; alert thresholds and configurations; data flows and interfaces; interoperability requirements; testing and validation procedures; maintenance, technical support, and contingency arrangements.

# 5. Organizational context

**Organizational context**

**Potential contribution**  If movement information is integrated into existing observation practices, care planning, handovers, and response routines, the system may support more coordinated and needs-based care rather than adding a parallel monitoring process.

**Questions and risks** Benefits are unlikely to emerge without clear criteria for when monitoring is used, defined responsibilities for reviewing information and responding to alerts, adaptation of workflows, leadership support, time for training, technical support, and alignment with documentation processes. Additional information may otherwise remain unused, duplicate existing observation practices, or create additional work.

**What to document** Criteria and workflows for initiating and ending monitoring; affected observation and response processes; responsibilities and escalation paths; integration into care planning, handovers, and documentation; staffing and resource implications; stakeholder involvement; implementation support; pilot scope; and review points.

| **Ethical and legal aspects** | **Cross-cutting reflection** Consider privacy and data protection, patient autonomy and consent, proportionality of monitoring, safety, transparency, equity, accountability, and applicable legal or regulatory requirements. In particular, consider whether monitoring is appropriate for the individual care situation, how patients are informed and involved, and how decisions are made when decision-making capacity is impaired.  **What to document** Document which movement-related data are collected and who can access them; how patients are informed and consent or objection is handled; how decisions are made when decision-making capacity is impaired; relevant safeguards and specialist reviews; responsibilities for monitoring and responding to alerts; and criteria for initiating, restricting, pausing, or discontinuing monitoring. |
| --- | --- |

# 6. Synthesis of key insights

Reflection across the domains reveals several interrelated tensions. These should not be resolved in the abstract but discussed with relevant stakeholders and documented in relation to the intended use, individual care situation, and local context.

| **Support for timely assistance vs. autonomy**  Potential support for recognizing situations requiring assistance must be balanced against privacy, dignity, autonomy, and individual control over monitoring. | **Standardization vs. individualization**  Defined criteria for initiating monitoring, configuring alerts, and responding to information can support consistent use, while monitoring decisions and responses still need to reflect individual needs and situations. |
| --- | --- |
| **Information vs. action**  Movement information and alerts create little value unless healthcare professionals can interpret them in context and respond appropriately. | **Potential vs. organizational reality**  Potential benefits depend on integration into existing workflows, adequate resources, clear responsibilities, technical support, and leadership support. |
| **Support vs. additional complexity**  Reduced uncertainty or improved situational awareness may be offset by frequent alerts, verification work, interruptions, and additional cognitive demand. | **Interoperability vs. isolated functionality**  Movement information that remains disconnected from existing information, communication, or documentation systems may limit usability, integration into routine practice, scalability, and sustainability. |

# 7. Provisional assessment

|  | **INTERPRETATION** The reflection does not support an unconditional implementation recommendation. It identifies conditions that should be clarified and tested before broader use. |
| --- | --- |

| **1** | **Purpose and target group**  Define which patients and situations the system is intended to support and when monitoring is not appropriate. |
| --- | --- |

| **2** | **Patient involvement and protection**  Establish transparent information and consent or representation processes, privacy and data protection measures, and procedures for involving patients or representatives in decisions about monitoring. |
| --- | --- |

| **3** | **Monitoring and response rules**  Specify criteria for initiating, adapting, pausing, and discontinuing monitoring; alert thresholds and configurations; responsibilities for reviewing information and responding to alerts; escalation paths; and the role of professional judgment. |
| --- | --- |

| **4** | **Technical and organizational fit**  Verify system performance, usability, interoperability, technical support, documentation requirements, and integration into existing observation, communication, and care workflows under local conditions. |
| --- | --- |

| **5** | **Evaluation**  Use a time-limited pilot with predefined indicators addressing implementation, usability and user experience, workflow and process effects, unintended consequences, and relevant care-related outcomes. |
| --- | --- |

# 8. Next steps beyond the NICT

|  | **BOUNDARY** The NICT structures reflection and supports the identification of relevant implementation conditions. The following activities build on its results to support decision-making and implementation planning but are not part of the tool itself. |
| --- | --- |

| **1** | **Clarify intended use and evidence needs**  Specify the intended purpose, target population, expected contribution, situations in which the technology should or should not be used, and relevant evidence gaps that need to be addressed. |
| --- | --- |

| **2** | **Assign responsibilities and decision structures**  Define who is responsible for implementation decisions, technical and organizational coordination, monitoring of the pilot, review of emerging concerns, and decisions about adaptation, continuation, or discontinuation. |
| --- | --- |

| **3** | **Define pilot and decision criteria**  Agree on the pilot scope, criteria for adaptation or discontinuation, relevant indicators and data sources, review points, and responsibilities for implementation decisions. |
| --- | --- |

| **4** | **Adapt workflows and responsibilities**  Define processes for monitoring and responding to information or alerts, documentation, escalation, communication, downtime procedures, and technical and organizational support. |
| --- | --- |

| **5** | **Prepare users and the organization**  Provide role-specific training, opportunities to practice using the system, accessible information, and mechanisms for feedback, questions, and reporting problems or unintended consequences. |
| --- | --- |

| **6** | **Evaluate and revisit**  Assess implementation, usability and user experience, workflow and process effects, unintended consequences, and relevant care-related outcomes. Repeat or update the NICT reflection when the technology, intended use, organization, or implementation context changes. |
| --- | --- |

|  | **DECISION POINT** Decide whether to stop, adapt, extend, or scale the implementation only after reviewing the documented assumptions, pilot findings, unresolved risks, and stakeholder perspectives. |
| --- | --- |
